# Creation of Artificial Cell-Like Structures Promoted by Microfluidics Technologies

**DOI:** 10.3390/mi10040216

**Published:** 2019-03-27

**Authors:** Yusuke Sato, Masahiro Takinoue

**Affiliations:** Department of Computer Science, Tokyo Institute of Technology, Kanagawa 226-8502, Japan

**Keywords:** microfluidics, water-in-oil emulsion, liposome, lipid vesicle, chemical communication, artificial cells, on-chip artificial cells, artificial molecular systems, molecular robotics

## Abstract

The creation of artificial cells is an immensely challenging task in science. Artificial cells contribute to revealing the mechanisms of biological systems and deepening our understanding of them. The progress of versatile biological research fields has clarified many biological phenomena, and various artificial cell models have been proposed in these fields. Microfluidics provides useful technologies for the study of artificial cells because it allows the fabrication of cell-like compartments, including water-in-oil emulsions and giant unilamellar vesicles. Furthermore, microfluidics also allows the mimicry of cellular functions with chip devices based on sophisticated chamber design. In this review, we describe contributions of microfluidics to the study of artificial cells. Although typical microfluidic methods are useful for the creation of artificial-cell compartments, recent methods provide further benefits, including low-cost fabrication and a reduction of the sample volume. Microfluidics also allows us to create multi-compartments, compartments with artificial organelles, and on-chip artificial cells. We discuss these topics and the future perspective of microfluidics for the study of artificial cells and molecular robotics.

## 1. Introduction

Answering the question “What is life?” is a persistent challenge in science [1,2]. In general, creating a system that is the same or similar to the system we want to understand can help us to deepen our knowledge of it. In this context, creating cell-like structures that mimic the functions of a living cell in a confined space (also known as artificial cells) may give us clues to answer the question. In addition, because artificial cells have a defined molecular composition, they can provide useful platforms to investigate biological phenomena under defined chemical conditions and can serve as tools for multiple biological applications, such as therapeutic techniques, fermentation by artificial cells, the construction of artificial red blood cells, and simplified cell models [3,4,5,6].

Biochemical reactions inside compartments surrounded by a cellular membrane or at the cellular interface play an important role in regulating many biological functions. In the study of artificial cells, cellular functions are mimicked or reconstructed using the same or similar biochemical components. In the past few decades, artificial cells exhibiting a part of cellular functions including DNA replication, translation, transcription, division, and morphological changes, have been developed using artificial compartments [7,8,9,10,11,12,13,14,15,16,17,18,19,20]. Many of the studies have employed water-in-oil (W/O) emulsions (or W/O droplet), polymersomes, or giant unilamellar vesicles (GUVs) as the compartments [7,8,9,10,11,12,13,14,15,16,17,18,19,20,21,22,23]. A W/O emulsion is a water drop in oil, which is covered with a monolayer of amphiphilic molecules such as artificial surfactants or lipid molecules. A polymersome is a vesicle comprising amphiphilic synthetic block copolymers. A GUV is a capsule structure formed by lipid bilayer membranes; it has been widely used as a compartment for artificial cells owing to its high similarity to the cellular membrane. Although these compartments can be constructed by simple methods, such as the mechanical agitation (sonication, pipetting, vortexing, or tapping) of a water drop in an oil, hydration of dry films of amphiphilic molecules, or emulsion phase transfer [24,25,26,27], microfluidics-based fabrication of such compartments can further accelerate the study of artificial cells because of its technological benefits.

Microfluidics is an interdisciplinary research field based on microfabrication technology, involving physics, chemistry, biology, and nanotechnology. It typically deals with small fluid volumes in a “chip” device or a tiny capillary [28,29,30]. In studies involving artificial cells, microfluidics provides a powerful means to efficiently produce a large number of compartments of a consistent size, within a scale ranging from a few dozen to several hundred micrometers. It is not easily achieved by traditional fabrication methods of the compartments. Furthermore, it enables the creation of multi-cellular compartments and the fabrication of compartments/reactors resembling living cells on the chip. These technological benefits in microfluidics will promote artificial-cell studies and lead to the design and construction of additional bio-inspired systems.

In this review, we introduce and discuss the microfluidics technologies that can produce artificial cells. We first outline typical methods for creating the compartments and newer methods with further advantages. Then, we describe chemical communication inside multi-compartments or among multiple compartments. We also describe unique studies with microfluidics, including artificial cells containing artificial organelles, living cells as organelles for artificial cells, and on-chip artificial cells. Finally, we discuss future perspectives in the field.

## 2. Microfluidic Fabrication of Compartments for Artificial Cells

### 2.1. Typical Fabrication Methods

Compartments with a defined size and producing a large number of the compartments allow investigations of biochemical reactions at defined volumes and efficient evaluations of the function of artificial cells. Microfluidics fabrication provides size controllability (several hundred nm to over hundreds µm) and a high-production rate (a few Hz to over thousands Hz) of the compartments.

**Figure 1:**
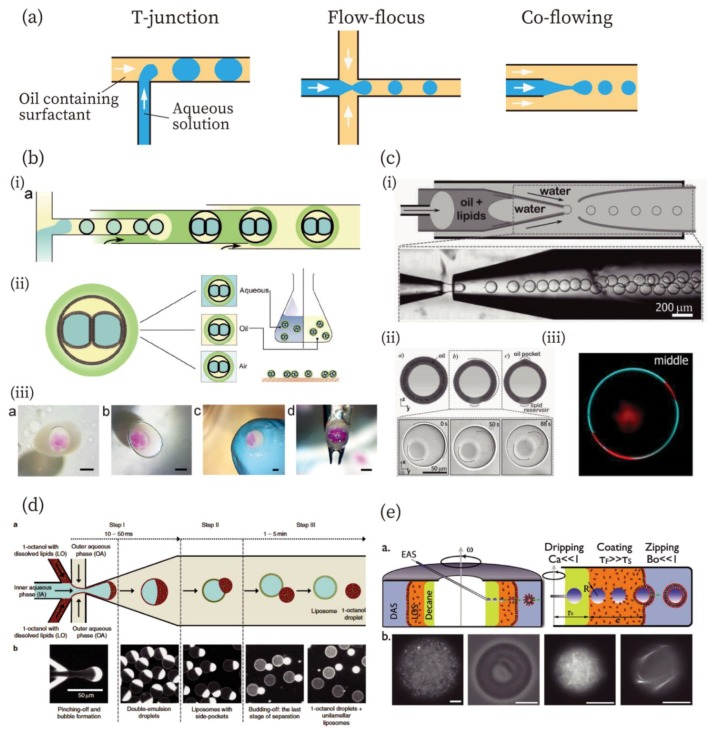
Methods for the production of compartments for artificial cells. (**a**) Typical design of flow channels for the fabrication of W/O emulsions: T-junction, flow-focusing, and co-flowing methods from left to right. (**b**) Droplet interface bilayers (DIBs) from the contact of two W/O emulsions in the large W/O emulsion covered with a gel-shell [43]. (i) Illustration of microfluidic fabrication of gel-encapsulated DIBs. (ii) Schematic illustrations of the gel-encapsulated DIBs in an aqueous, an oil, and an air phase. (iii) Gel-encapsulated DIBs in aqueous, oil, and air phase and manipulated using a tweezer. Scale bars: 1 mm. Reproduced with permission from [43]. (**c**) Fabrication of giant unilamellar vesicles (GUVs) with an ultrathin oil shell using the double emulsion method [49]. (i) An illustration and image for the fabrication of double emulsions with an ultrathin shell. (ii) Illustrations and microscopic images of GUVs with ultrathin shells. (iii) Phase separation of the GUV prepared by this method. Red and cyan colors indicate liquid-disordered and liquid-ordered phases, respectively. Reproduced with permission from [49]. (**d**) Fabrication of GUVs by the octanol-assisted method [50]. Reproduced with permission from [50]. (**e**) A continuous droplet interface crossing encapsulation (cDICE) method for GUV fabrication [52]. Scale bars: 10 µm. Reproduced with permission from [52].

The methods used to create W/O emulsions using microfluidic devices were developed in the 2000s: the T-junction method [31], the flow-focus method [32], and the co-flowing method [33,34] (Figure 1a). All methods are categorized as droplet microfluidics technologies. Two immiscible phases, an aqueous phase containing components for artificial cells and an oil phase with surfactant molecules, flow in channels at designated flow rates. By adjusting these flow rates, W/O emulsions can be formed at a chosen size as a product of shear stress by the oil flow. Viscosity of oil and interfacial tension between two immiscible solutions are also important parameters for fabrication of the emulsions. Readers can gain a further understanding of the droplet microfluidics in previous review articles [30,35,36,37], in which the usage of droplet microfluidics is described in more detail. In this section, we introduce typical methodologies employed to fabricate the compartments for artificial cells based on the droplet microfluidics.

Droplet microfluidics technology can be used to fabricate not only W/O emulsions, but also other compartments for artificial cells, including multi-component double emulsions [38,39,40], droplet interface bilayers (DIBs) [41,42,43,44,45,46], polymersomes [26,47], and GUVs [48,49,50,51,52,53,54,55,56,57,58]. In the case of multi-compartment double emulsions, smaller emulsions are further encapsulated into larger emulsions. The methods reported by Adams et al., Che et al., and Chu et al. allow the encapsulation of a large number of smaller, inner emulsions into the outer emulsion [38,39,40]. Regarding DIBs, Elani et al. and Walsh et al. have reported DIBs in a W/O emulsion containing multiple droplets [41,42]. In addition, Baxani et al. have developed a robust platform for creating artificial cells with a DIB [43], combining the T-junction and co-flowing methods (Figure 1b). The chassis is covered with an alginate gel compartment containing droplets that formed the DIB. The chassis was found to be stable in an aqueous, an oil, and even in an air phase, and was also mechanically stable (Figure 1b (ii) and (iii)).

Polymersomes and GUVs are fabricated by similar approaches [26,51]. In this review, we focus on GUVs more than on polymersomes because of the higher similarity of GUVs to cellular membranes. Researchers have developed multiple fabrication methods for GUVs, primarily W/O/W double emulsion-templated [48,49,50,51], emulsion-transfer [52,53,54,55,56], and jetting methods [57,58].

**Figure 2:**
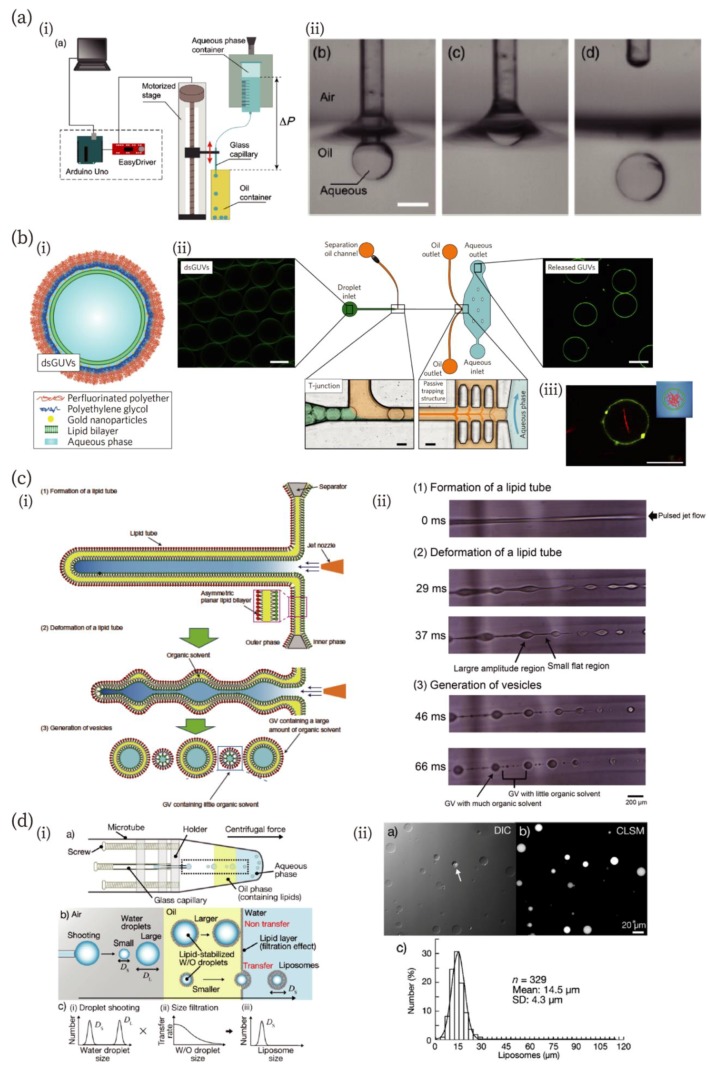
Novel and unique microfluidic technologies improving the fabrication of artificial cell-compartments. (**a**) A low-cost capillary-based fabrication method of W/O emulsion [66]. (i) An illustration of the low-cost setup. (ii) Snapshots of processes of droplet fabrication. Scale bar: 400 µm. Reproduced with permission from [66]. (**b**) A microfluidic chip for the fabrication of droplet-stabilized giant unilamellar vesicles (dsGUVs) and for the release of GUVs from the droplet [67]. (i) A schematic of the dsGUV. (ii) The microfluidic device used to release the GUVs from the surrounding stabilizing polymers into an aqueous phase. (iii) A fluorescence microscopic image of GUVs encapsulating actin filaments (red). Scale bars: 20 µm. Reproduced with permission from [67]. (**c**) A jetting-based fabrication method for asymmetric GUVs containing little organic solvent in the membrane [71]. (i) and (ii) Schematics and microscopic images of the formation process, reproduced with permission from [71]. (**d**) Droplet-shooting and size-filtration (DSSF) method for the fabrication of the compartment [73]. (i) Schematics of the device and formation process of GUVs. (ii) Differential interference contrast (DIC) and confocal laser scanning microscopic (CLSM) images of GUVs, and size-distribution of GUVs. The white arrow in the DIC image indicates a multilamellar vesicle. Reproduced with permission from [73].

The double emulsion-templated method was developed by Shum et al. in 2008 [48]. The double emulsions were generated using a microcapillary fluidic device that combines the co-flowing and the flow-focus methods. Arriaga et al. have reported GUVs with ultrathin oil shells using the double emulsion method (Figure 1c) [49]. A double emulsion with an ultrathin shell was created based on previous work [59] by the same group (Figure 1c (i)). The ultrathin shell was achieved by exploiting the affinity of the fluid for the glass capillaries. The double emulsions with ultrathin oil shells were converted into GUVs with the formation of an oil pocket and lipid reservoir by the evaporation of organic solvents (Figure 1c (ii)). After the formation of the unilamellar membrane, the GUVs exhibited phase separation of the membrane (membrane composition: DOPC/DPPC/cholesterol = 35:35:30 mol%) (Figure 1c (iii)). Using a similar approach, Deshpande et al. have demonstrated that GUVs were spontaneously formed from double emulsions (Figure 2d) [50]. They used 1-octanol as the oil phase for lipids. The 1-octanol oil pocket was formed in lipid vesicles, but it completely budded off from the double emulsions owing to the minimization of interfacial energy, resulting in the formation of GUVs.

An emulsion transfer method was first reported by Pautot et al. in 2003 using a test tube and a centrifuge, which enabled them to fabricate GUVs with asymmetric bilayer membranes [60,61]. This technique has been expanded to microfluidics to prepare GUVs. Abkarian et al. have developed a continuous-droplet interface-crossing encapsulation (cDICE) method (Figure 2e) [52], consisting of a glass capillary and a centrifuge cylindrical chamber with a hole in the lid. Dispersing aqueous solution (DAS), lower density lipid-in-oil solution (LOS) (mineral oil), and decane were poured into the rotating-cylindrical chamber in this order. The encapsulated aqueous solution (EAS) was injected from the capillary in the decane phase. W/O emulsions were formed in the decane and moved to the LOS phase, where they were covered with a lipid monolayer. GUVs were formed when the W/O emulsions passed through the LOS/DAS interface, where a lipid monolayer was added. Detailed characteristics of the cDICE methods were further investigated by Blosser et al. [53]. In addition to the cDICE method, Hu et al. have reported GUV production using emulsion transfer, in which W/O emulsions were fabricated using microfluidics and then poured onto a lipid monolayer at an oil/water interface [54]. Furthermore, Matosevic et al. and Karamdad et al. have reported microfluidic devices that can produce GUVs from W/O emulsions in a single microfluidic chip using emulsion transfer [55,56].

In the jetting method, vesicles were generated by applying a pulse jet flow against a planer lipid bilayer from a small glass capillary [57,58]. The pulse jet induced prominence of the lipid bilayer and the prominence was detached from the bilayer, resulting in the formation of vesicles. In addition to the methods described above, other approaches have also been investigated. For example, Ota et al. developed a unique method to generate GUVs [62]. Lipid bilayers were formed in a small microfluidic chamber that was connected to a large chamber. Outward flow from the large to the small chamber was generated by the irradiation of a laser to the large chamber. The bilayer-covered solution was pushed from the small chamber and shear forces from a continuous fluid stream led to the fission of the bilayer-covered solution, resulting in the generation of GUVs. In a method reported by Tan et al., W/O lipid emulsions in oleic acid prepared by a microfluidic device were poured in an ethanol/water mixture [63]. The oleic acid dissolved in ethanol, and lipids were forced to rearrange around the emulsion to assemble into GUVs. Matosevic et al. developed a layer-by-layer approach in which W/O emulsions trapped in the microfluidic chamber were further covered with a lipid monolayer [64]. It resulted in the formation of GUVs and allowed multilamellar vesicles to form. Readers can find more details of microfluidic-fabrication methods for lipid vesicles in a review article [65]. In the methods described in this section, microfluidic fabrication of the compartments for artificial cells contributes advantages including highly efficient production, monodispersity, size-controllability, and well-encapsulated yields of components into the inner aqueous phase. 

### 2.2. Novel Microfluidic Fabrication Methods for Compartments with Additional Advantages

In the past few years, attempts to improve microfluidics technologies have followed several routes: simplicity at a low cost, stability of the compartments during the fabrication process, asymmetricity of the lipid bilayer without an organic solvent, and reduction of the consumed sample volume. Here, we summarize recent studies improving microfluidics technologies for artificial cell studies.

Valet et al. developed a simple method to produce aqueous droplets in an oil, which they named the “capillary-trap method” (Figure 2a) [66]. In their device, a glass capillary is cyclically moved up and down across the oil-air interface (left in Figure 2a). The capillary is immersed in the oil and an aqueous droplet is formed by flowing the aqueous solution in the capillary. When the capillary is vertically pulled out, the droplet detaches from the capillary because of the minimization of surface energy in the oil-water-air phase (right in Figure 2a). This method produced droplets of a minimum radius of approximately 30 µm. The authors stated that this method can produce about 1 µm droplets in principle. In addition to the development of the method, the authors also developed a low-cost and easy-to-implement setup for their technique. According to the authors’ evaluation, the maximum cost is about a few hundred euros [66].

Although GUVs are often used due to their similarities to biological membranes, their chemical and mechanical instabilities make the creation of complex and functionalized artificial cells difficult. Weiss et al. addressed these points by developing a microfluidic method to generate stable GUVs termed “droplet-stabilized giant unilamellar vesicles (dsGUVs)” [67] (Figure 2b). In their method, a continuous lipid bilayer is formed at the inner interface of an amphiphilic block/copolymer-stabilized water-in-oil droplet. Multiple biomolecules can be loaded into dsGUVs using picoinjection [68]. The authors have demonstrated the loading of transmembrane proteins (F_1_F_o_ ATP-synthase and integrin) and cytoskeletal proteins (G-actin and tubulin) into dsGUVs. In addition, the authors showed that GUVs in stabilizing polymer droplet shells can be released into an aqueous phase from the oil phase. They designed a microfluidic chamber with passive trapping structures. The dsGUVs are separated from each other at the T-junction and flow in the oil with destabilizing surfactants; then, the dsGUVs are decelerated at the trapping structures and gradually approach the oil-water interface. When the destabilized droplets contact the interface, GUVs are released into the aqueous phase. This release mechanism can be applied to dsGUVs containing actin filaments (bottom right in Figure 2b) and integrin.

Eukaryotic cellular membranes have asymmetrical inner and outer leaflets. This asymmetry is hypothesized to play an important role in biological processes [69,70]. Therefore, artificial cells with asymmetric lipid bilayers can serve as a platform to investigate the role of the asymmetricity in living cells. Pautot et al., Hu et al., and Funakoshi et al. have created asymmetric GUVs using microfluidics; however, in their methods, a large amount of organic solvents remained in the bilayer, which will affect important membrane dynamics, such as lipid flip-flop phenomena. Kamiya et al. developed a method to fabricate asymmetric GUVs containing little organic solvent in their membranes [71] with a pulse-jetting method [57] (Figure 2c). They prepared a planar asymmetric lipid bilayer membrane using a droplet contact method [72], and microfluidic flow was applied to the membrane, resulting in the formation of a lipid tube (Figure 2c (i)). They found that large (100–200 µm) and small (3–20 µm) GUVs were formed during the deformation process of the lipid tube due to Rayleigh–Plateau instability (Figure 2c (ii)). Using a Raman scattering microscope, they showed that small GUVs with the asymmetric bilayer contained only a very small amount of organic solvents. They also investigated the time-course of flip-flop dynamics of lipid molecules and influence of peptides on the flip-flop. 

Microfluidics generally requires a large amount of sample solution because of tubing connected to a microfluidic chamber, adjustment of the flow rate, or preparing steady flow. This is an important issue since artificial cells often include rare and expensive reagents. Thus, reduction of the sample volume is one of the important issues in microfluidics. A novel method for the production of GUVs (or W/O emulsion) in extremely small sample volumes (0.5 µL) has been devised by Morita et al. [73]. They fabricated a centrifugal capillary-based microfluidic device employing the W/O emulsion transfer method. Water microdroplets were discharged from the tip of a glass capillary toward an oil phase containing lipids, and the lipids stabilized the W/O emulsion transfer through an oil-water interface, creating GUVs (Figure 2d). Although large (~100 mm) and small (~15 µm) droplets were discharged from the tip, only cell-sized GUVs were formed due to the size-filtration effect. The detailed mechanism is described in the article. They called this method “droplet-shooting and size-filtration” (DSSF). This method allows the production of hundreds of GUVs from a single microliter of sample without wastage. Such low-sample volume microfluidic techniques will further accelerate the production of artificial cells.

## 3. Communication Among Artificial Cells

Intercellular chemical communication, in which secreted molecules are diffused or are transported to other cells, regulates multiple cellular functions, including differentiation and morphogenesis [74,75]. This has important roles, not only in multicellular organisms, but also in the collective behavior of single-celled organisms [76]. Therefore, communication between artificial cells and communication between artificial cells and living cells has the potential to lead to the construction of artificial tissue structures, artificial cells capable of controlling their function based on individual interactions, and “development of life like technology” [77]. Lipid vesicle networks connected via lipid nanotubes [78,79] and a multi-compartment of W/O emulsions [80] have been fabricated by manual manipulation. Moreover, microfluidics has the potential to automate the fabrication of multicellular structures with a large number of single compartments containing arbitrary molecules in each compartment [81,82,83,84]. In this section, we describe chemical communication among artificial cells based on microfluidics technology.

**Figure 3:**
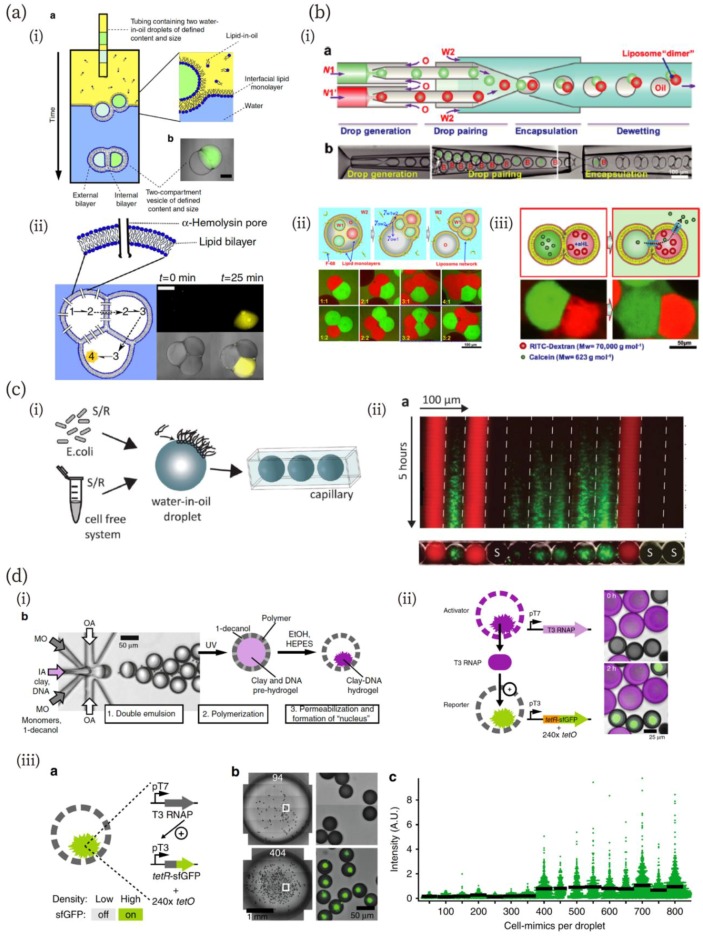
(**a**) Multi-compartment formation via the phase transfer of multiple droplets and cascadic chemical reaction in the compartment [86]. (i) Schematics of the fabrication method and a microscopic image of a multi-compartment vesicle. (ii) Three-step cascadic-chemical reaction in the multi-compartment. Scale bar in microscopic images: 250 µm. Reproduced with permission from [86]. (**b**) Multi-compartment GUVs and selective molecular release from them [87]. (i) Schematics and microscopic images of the fabrication process of the multi-compartments via dewetting of W/O emulsions. (ii) Schematics and microscopic images of the multi-compartments consisting of two, three, four, and five compartments. (iii) Size-dependent molecular release from the multi-compartment to an outer solution through alpha-hemolysin pores. Reproduced with permission from [87]. (**c**) Chemical communication between a cell-free system and bacteria [92]. (i) Schematics of the experimental system. (ii) A kymograph of a fluorescence microscopic image of chemical communication between cell-free and bacteria droplets. Reproduced with permission from [92]. (**d**) Quorum sensing (QS) of artificial cells [95]. (i) Microfluidic fabrication of artificial cells capable of gene expression in the nuclei. (ii) Protein exchanges between neighboring artificial cells. (iii) QS of artificial cells with fluorescence accumulation, depending on the artificial cell density. Reproduced from [95].

Elani et al. have reported a construction method for multi-compartment vesicles based on the W/O emulsion [85,86]. Multiple W/O droplets were tandemly loaded in a single tube and deposited onto interfacial lipid monolayers from the oil phase, resulting in the formation of a multi-compartment through phase transfer (Figure 3a (i)) [86]. The authors demonstrated a cascading chemical reaction in this system, in which each reaction step was isolated in a distinct compartment by a lipid bilayer (Figure 3a (ii)). The dewetting phenomenon of W/O/W double emulsions can also be used to fabricate multicellular structures [83,87]. Deng et al. have developed a method for assembling multicompartment vesicles from a double emulsion (Figure 3b) [87]. The double emulsion was fabricated using a microfluidic device combining co-flow and flow-focusing methods (Figure 3b (i)). The oil phase and outer aqueous phase (W2 in Figure 3b) contain egg L-α-phosphatidylcholine and a triblock copolymer, Pluronic F-68. They demonstrated that the spontaneous dewetting process of double emulsions can be controlled by adjusting the interfacial tension between aqueous and oil phases, utilizing a triblock copolymer to ensure complete dewetting of the double emulsion (Figure 3b (ii)). The authors also demonstrated molecular transfer to the outer aqueous solution through the multi-compartments (Figure 3b (iii)). They loaded small and large fluorescent molecules (calcein and RTIC-Dextran, respectively) to each compartment, where αHL pores were formed at the one compartment loading large molecules. As a result, small molecules passed through to another compartment and eventually diffused to the outer solution, while the large fluorescent molecule was retained. H. Bayley’s group successfully constructed a huge tissue-like multicompartment by printing a W/O emulsion [88,89,90,91]. They demonstrated that each compartment comprising the artificial tissue can communicate with others via pores. 

In addition to communications between artificial molecular components, chemical communication between a cell-free system and living organisms (bacteria) has been achieved (Figure 3c) [92]. Schwarz-Schilling et al. used amphiphilic molecules of N-acyl-l-homoserine lactones (AHLs) and isopropyl-β-d-thio-galactopyranoside (IPTG) as communication signals among W/O emulsions. They designed a genetic AND gate that responded to AHL and IPTG, leading to the expression of the green fluorescent protein (GFP) and a sender circuit that produced AHL when stimulated by the IPTG signal. The AND gate or sender plasmid was cloned in Escherichia coli (*E. coli*) or mixed with a cell-free gene expression system. The *E. coli* and the cell-free system were encapsulated into separate droplets, which were then loaded into a capillary (Figure 3c (i)). They demonstrated communication between the cell-free system and bacteria using their AND gate and sender circuits (Figure 3c (ii)). IPTG, cell-free sender circuits, and the bacteria AND gate were encapsulated into droplets, which were shown with red, no-color (shown as “S” in the figure), and green color markers, respectively. In this system, IPTG diffused to both bacterial and the sender-circuit droplets. The sender droplets that received IPTGs produced AHLs and the produced AHLs diffused to other droplets, including the bacterial droplets. When IPTGs and the produced AHLs diffused into the bacterial droplets, the AND gate was switched and the bacteria expressed GFP. Furthermore, the direction of communication could be reversed by encapsulating bacteria with sender circuits and the cell-free system with the AND gate into droplets. The GFP expression level depended on the distance from IPTG and sender droplets because the concentration of diffused molecules in droplets depends on their distance from the diffusion source [92].

Quorum sensing (QS) is a common mode of chemical communication in bacteria for controlling specific gene transcription and collective behavior, depending on population density [93]. Since microfluidics can generate a large number of cell-like compartments of controlled sizes, it enables the mimicking of QS-type chemical communication [94,95]. Niederholtmeyer et al. have shown this using artificial nuclei (Figure 3d) [95]. They fabricated artificial cells with a flow-focusing method (Figure 3d (i)). These artificial cells had clay-DNA nuclei with genetic information. A porous polymer membrane allowed the introduction of large molecules, such as cell-free transcription/translation reagents, from the outside to inside. The authors demonstrated that the artificial cells exchanged proteins with their neighbors (Figure 3d (ii)). They prepared activator and reporter artificial cells containing gene templates for T3 RNA polymerase (RNAP) and for the T3 RNAP-driven synthesis of the TetR-sfGFP reporter, as well as a tetO array plasmid. The T3 RNAPs produced from the activator were successfully transferred to the reporter, which exhibited green fluorescence. By using a similar technique, the authors produced artificial QS (Figure 3d (iii)). The artificial cells containing both the activation and reporter constructs exhibited fluorescence as a function of density. The threshold density they observed was 400 artificial cells in 4.5 µL volume. 

## 4. Artificial Organelle in Artificial Cells

**Figure 4:**
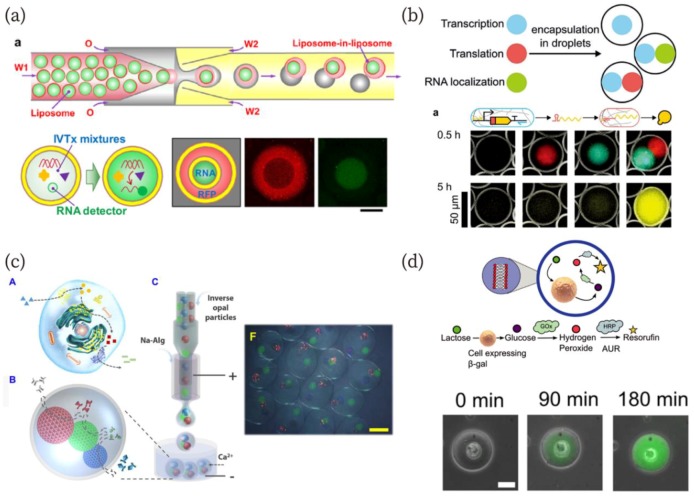
(**a**) Schematics of the fabrication method for “liposome-in-liposome”, and in vitro transcription and transcription/translation in “artificial nuclei” and “cytoplasm” [96]. Scale bar: 50 µm. Reproduced with permission from [96]. (**b**) Artificial gel-based organelles in W/O emulsion that exhibit transcription and translation in each artificial organelle [98]. Reproduced with permission from [98]. (**c**) Schematics of a biomimetic enzyme cascade reaction in the microcapsule and a microscopic image of the microcapsule with three-cores [99]. Scale bar: 400 µm. Reproduced with permission from [99]. (**d**) A schematic of the enzymatic pathway containing a living cell as an organelle-like bioreactor and sequential microscopic images representing a successful reaction with the aid of the cell [100]. Reproduced with permission from [100].

In a typical approach toward the creation of artificial cells, components are mixed in a batch reaction solution; however, living cells spatially divide functional modules as organelles including the nucleus, Golgi apparatus, endoplasmic reticulum, and others. Construction of functional modules in a cell-mimicking compartment is a promising strategy for the realization of artificial cells. Many researchers have demonstrated the usefulness of microfluidics for the creation of artificial organelles in artificial cells [96,97,98,99,100,101,102]. Deng et al. fabricated liposome-in-liposomes (vesosomes) using microfluidics and the dewetting mechanism [96]. The inner and outer liposomes in the vesosomes contain an in vitro translation system and in vitro translation/transcription system, respectively (Figure 4a). The liposome successfully translated RNA only in the inner, “nuclear” liposome and expressed monomeric red fluorescent protein (mRFP) in the outer, “cytoplasmic” liposome. Multiple artificial organelles in artificial cell models have also been demonstrated. L. Aufinger and F. C. Simmel developed hydrogel-based artificial organelles (Figure 4b) [98]. They immobilized gene-length DNA in agarose gels and prepared the gel-based artificial organelles using microfluidics. The organelles were equipped with different functions: gene transcription, translation, and RNA localization. They investigated each artificial organelle function and demonstrated translation and transcription by the organelles in W/O droplets. This gel-based organelle fabrication approach can provide enzymatic cascade reactions via each organelle. Wang et al. have recently reported a multienzyme system with this feature in hydrogel microcapsules [99]. They immobilized enzymes in inverse opal particles (IOPs), which were encapsulated into a hollow alginate hydrogel (Figure 4c). The multienzyme cascade reaction was demonstrated using horseradish peroxidase, ß-glucosidase, and glucose oxidase, which were immobilized in different IOPs in the capsule. As reaction substrates, octyl ß-D-glucopyranoside, o-dianisidine, and hydrogen peroxide were used. Detailed cascade reaction pathways were described in the article [99]. Interestingly, this cascade reaction within the microcapsule exhibited a higher performance than in the absence of encapsulation. The authors explained that this was due to the shorter distance between the IOPs in the capsule. 

The use of living cells as organelles for artificial cells has also been reported [100]. Encapsulation of living cells into a compartment such as a W/O droplet has been widely studied because such techniques can be used for the high-throughput screening of cells [103,104,105]. Elani et al. constructed GUVs with embedded living cells as organelles [100]. The authors encapsulated living cells into GUVs using a microfluidics technique, and the embedded cells served an organelle-like function in a cascading enzymatic reaction (Figure 4d). The reaction was demonstrated in a hybrid cellular bionic system. In addition to the organelle-like behavior, they showed cell growth in the GUVs. Morita et al. also showed bacterial growth in GUVs fabricated by a microfluidic device [101]. These results pave the way for the creation of living cell-artificial cell hybrid organelle systems. 

## 5. On-chip Bio-inspired Molecular System

In the above sections, we described artificial-cell studies based on cell-mimicking compartments fabricated using microfluidic devices. Microfluidics can also provide a confined space for mimicking biological reactions. Therefore, a combination of biochemical reactions and microfluidic devices can produce artificial cells that work “on-chip”. In fact, some researchers have created such artificial cells or cell-mimicking biochemical reactions in microfluidic channels. 

**Figure 5:**
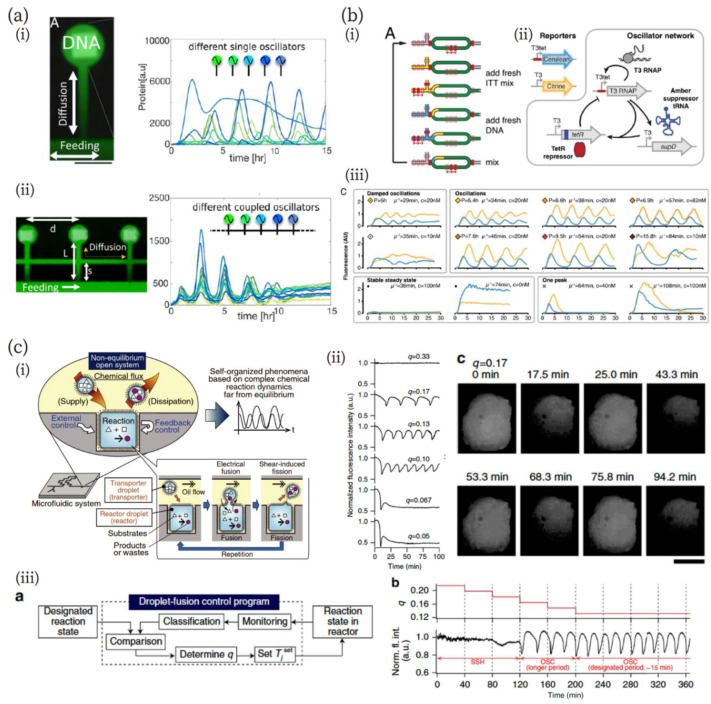
(**a**) On-chip DNA compartments as artificial cells [106]. (i) An image of expressed green fluorescence protein (GFP) (green) and DNA patterns (white), and a graph of oscillation dynamics in 15 different compartments. Scale bar: 100 µm. (ii) An image of three coupled compartments and a graph of the synchronized oscillations in the coupled compartments. Reproduced with permission from [106]. (**b**) In vitro transcription/translation system in a microfluidic nanoreactor [113]. (i) and (ii) Schematics of the nanoreactor (i) and an oscillator network (ii). (iii) Oscillation behavior of the network in the nanoreactor controlled by microfluidic parameters. Reproduced with permission from [113]. (**c**) Chemical reactions far from equilibrium in a droplet open-reactor system [114]. (i) Schematic diagrams of the experimental system in which fusion/fission of droplets were electrically controlled. (ii) pH Oscillation reaction depending on the ratio of fusion states in the fusion–fission process (shown as *q* in the figure). Scale bar: 500 µm. (iii) Feedback control of pH oscillation using the droplet open reactor system: scheme of feedback control (left) and time course of *q* and oscillation behavior during the feedback control. Reproduced with permission from [114].

Bar-Ziv et al. created on-chip biological systems capable of mimicking metabolism, programmable protein expression, communication, synchronization, and pattern formation [106,107,108]. In their system, DNA-encoding genetic networks were patterned on the surface of circular compartments carved in silicon (Figure 5a) [106]. The DNA compartments were connected to a main channel in which cell-free extracts flowed via a narrow diffusive channel. This design allowed gene-network dynamics to be controlled, depending on the compartment geometry and biological reaction; i.e., controlling reaction-diffusion dynamics. The constructs for an oscillatory gene network were DNA in compartments. The authors demonstrated the oscillation of protein expression in 15 different compartments (Figure 5a (i)). Furthermore, to couple the oscillations, the authors connected each compartment at an appropriate geometry, which allowed the diffusive transportation of newly synthesized proteins (Figure 5a (ii)). As a result, in the 15 compartments, the frequency and phase of protein expression were successfully synchronized (Figure 5a (ii)). This research group has also reported the propagation of gene expression on similar on-chip artificial cells [107]. They designed a one-dimensional array of DNA compartments with a bistable genetic circuit for propagation. They showed front propagation of gene expression along the artificial cell arrays based on reaction-diffusion dynamics and investigated the behavior of the gene circuit at monostable, bistable, and transition points. Such reaction-diffusion dynamics have an important role in pattern formation during development. The on-chip biological system exhibited the pattern formation of gene expression [106]. In addition, Zadorin et al. have shown a French flag pattern formed by a DNA chemical reaction network in a flow channel [109]. The reaction network comprised the PEN DNA tool box [110]. They demonstrated that two orthogonal bistable chemical reaction networks could produce a French flag pattern of DNA concentration in the flow chamber. 

Besides reaction-diffusion dynamics on a chip, electrical control of microfluidic channels provides a powerful means to investigate and mimic biochemical reactions [111,112,113,114,115]. Niederholtmeyer et al. have developed a microfluidic device in which an in vitro translation/transcription system functions at a steady state by exchanging the solution via multi-flow channels and computer-controlled valves (Figure 5b) [113]. The microfluidic device has circular reactors, with the inlet and outlet controlled by a computer (Figure 5b (i)). They confirmed that their device can keep the system at a steady state and that the concentration of each reaction component can be controlled by regulation parameters, including replaced-volume and dilution rate and residence time. The authors designed a biological oscillator network (Figure 5b (ii)) and investigated its oscillation behavior. As they expected, steady-state conditions were necessary to produce oscillations. The oscillator network exhibited multiple states: damped oscillations, a stable steady state, oscillations with different periods, and one peak pulse (Figure 5b (iii)). 

Sugiura et al. have addressed chemical oscillation far from equilibrium using a droplet open-reactor system in a microfluidic system (Figure 5c) [114]. Their method is similar to the molecular transportation systems in living cells; namely, chemical fluxes were controlled by the fusion/fission of transporter droplets regulated by electrical stimulation (Figure 5c (i)). The detailed fusion/fission methods for the reactor system were described in the previous article [116]. The authors investigated the controllability of chemical reaction dynamics far from equilibrium using the system of bromate-sulfite-ferrocyanide pH oscillation [117]. They confirmed that this reaction generated pH oscillation when chemical fluxes were maintained appropriately (Figure 5c (ii)). Furthermore, autonomous feedback control of the chemical reaction was demonstrated using a feedback control system (Figure 5c (iii)). The state of the reactor was monitored and classified, and then, the fusion/fission interval of the transporter was changed to the designated one. As shown in the figure, the state of the chemical reaction was successfully adjusted to the oscillation state from the steady state in the droplet open-reactor system.

## 6. Conclusions and Future Perspectives

In this review, we have described microfluidics as a powerful technology for the study of artificial cells, particularly for the creation of compartments for artificial cells because of its high production efficiency and size-controllability. Although there are still significant differences between the living cellular membrane and artificial compartments, microfluidics techniques have the potential to fill the gap. As we described in this review, microfluidics provides methods for the fabrication of oil-less GUVs, asymmetric membranes, and protein-embedded compartments. These methods will lead to the creation of compartments more similar to living cells. We have also noted that microfluidics enables us to fabricate multi-compartments, liposome-in-liposomes, and compartments containing artificial organelles. The contribution of microfluidics techniques to fabricating multi-compartments containing artificial organelles will be key for the construction of artificial tissues, which is one of the practical applications of artificial cells. The studies of on-chip artificial cells described in this review aimed to mimic cellular functions. Although there are some differences at the boundary between the on-chip systems and the vesicle-based systems, such as the diffusion of molecules on the surface of the boundary, covering the chip with biological membranes (lipid monolayers or supported lipid bilayers) will fulfill the gap and expand the applicability of the on-chip artificial cells. Because therapeutic applications of artificial cells have been proposed [3], on-chip artificial cells are likely to be applicable for biomedical devices, such as diagnosis, therapeutic, or monitoring devices. 

**Figure 6:**
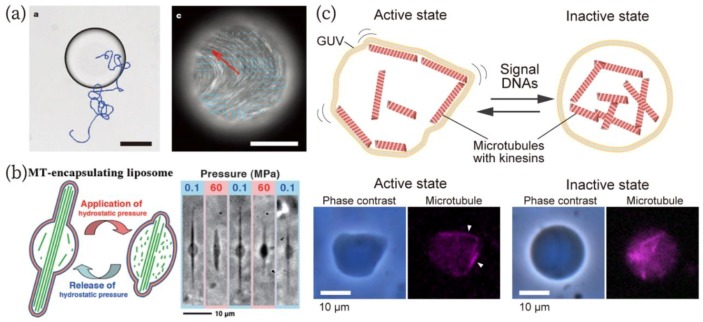
(**a**) Autonomous motility of the W/O droplet containing microtubule-kinesin bundles [120]. A bright-field image of the droplet (left) and a fluorescence image of microtubules (right). Scale bars: 80 µm (left) and 100 µm (right). Reproduced with permission from [120]. (**b**) Morphological changes of GUVs encapsulating tubulins that are polymerized to microtubules in response to hydrostatic pressures [14]. Reproduced with permission from [14]. (**c**) An amoeba-type molecular robot capable of changing its shape in response to signal DNA molecules [127]. Reproduced with permission from [127].

A parallel research field to that of artificial cells has recently emerged, called molecular robotics. In this field, researchers aim to create artificial molecular systems comparable to or outperforming the corresponding natural living system [118,119]. The realization of conformational changes and locomotion are two of the major targets in this field. From the perspectives of the reconstruction of cells and molecular robotics, researchers have so far reported locomotion and shape changes of W/O droplets [120,121,122], oil droplets in water [123], and GUVs [12,13,14,15,124,125,126] (Figure 6a,b). In 2017, the robotic behavior of GUVs, as in sensing signal molecules, processing the signals, and exhibiting actuation, was achieved. Sato et al. showed an amoeba-type molecular robot that changes shape in response to signal molecules produced inside the robot via photo-irradiation (Figure 6c) [127].

As described above, GUVs are utilized as a chassis for the molecular robot [119,127,128]. Therefore, microfluidics will also contribute to the creation of the chassis for molecular robots, as well as artificial cells and their subcompartments. In addition, we expect two additional contributions of microfluidics to molecular robotics. The first is as an assembly factory. The pico-injection method [68] will allow us to sequentially assemble robot parts in the intended order in the W/O emulsions (GUV precursors) flowing in microfluidic channels. Sorting techniques for a single cell in microfluidic devices [129] can serve as a quality control system for removing misassembled robots. Thus, microfluidic devices with injectors and sorters could make a large contribution to the development of molecular robotics. The second is the fabrication of testing grounds to evaluate a robot’s function. Potential applications of molecular robots are in biomedical and pharmacological fields [118,119]. Microfluidic cell-culture systems [130] and blood-vessel mimics on a chip [131] would be applicable as testing grounds (mimics of inside the body) for molecular robots having medical functions to investigate the efficacy of drug transport to the cells, to study the effects of the stimulation of cells by robots, and others. Such uses of microfluidics can also be adapted to the future application of artificial cells in biomedical fields.

In conclusion, microfluidics technology has clearly promoted the study of artificial cells. Although the early stage of microfluidics only provided cell-sized compartments, the great progress in the past years allows us to more closely study artificial cells. The progress has accelerated and contributed to the development of biological and engineering fields. We believe that microfluidics will become an indispensable technology from fundamentals to applications in multiple fields, including synthetic biology, artificial cell studies, and molecular robotics.
